# Examining factors for the adoption of silvopastoral agroforestry in the Colombian Amazon

**DOI:** 10.1038/s41598-023-39038-0

**Published:** 2023-07-28

**Authors:** C. O. Alvarado Sandino, A. P. Barnes, I. Sepúlveda, M. P. D. Garratt, J. Thompson, M. P. Escobar-Tello

**Affiliations:** 1 Rural Economy, Environment and Society, SRUC, The Kings Buildings, West Mains Road, Edinburgh, UK; 2 Faculty of Geosciences, University of Edinburgh, West Mains Road, Edinburgh, UK; 3 Sustainable Land Management, School of Agriculture, Policy and Development, University of Reading, Reading, UK; 4 UK Centre for Ecology and Hydrology, Bush Estate, Penicuik, UK; 5 Bristol Veterinary School, University of Bristol, Langford House, Bristol, UK

**Keywords:** Agroecology, Climate-change adaptation, Climate-change mitigation, Psychology and behaviour

## Abstract

Current land use systems in the Amazon largely consist of extensive conventional productivist livestock operations that drive deforestation. Silvopastoral systems (SPS) support a transition to low carbon production if they intensify in sympathy with the needs of biophysical and socio-economic contexts. SPS have been promoted for decades as an alternative livestock production system but widespread uptake has yet to be seen. We provide a schema of associating factors for adoption of SPS based on past literature in tropical agriculture and apply this to a bespoke survey of 172 farms in the Caquetá region of the Colombian Amazon. We find a number of factors which do not apply to this region and argue for a context specific approach. The impact of managing increased market access and opportunities for SPS producers are crucial to avoiding additional deforestation. Further understanding of the underlying antecedents of common factors, such as perceptions of silvopastoral systems, would reduce the risk of perverse policy outcomes.

## Introduction

Deforestation and agricultural expansion endanger the functioning of the Amazon ecosystem and the livelihoods and wellbeing of the communities who live from this resource^1^. A reinforcing feedback cycle emerges from the coupling of poor physicochemical soil quality with unsustainable ranching that drives further degradation, eventually forcing farmers to abandon their unproductive land in search of native forest to colonise, thus restarting the degradation cycle^2,3^. Silvopastoral systems (SPS) offer an alternative to conventional ranching systems^4^. Generally, a SPS incorporates perennial trees and shrubs into pastures to reflect some of the ecosystem services provided by native forests while providing more consistent and higher quality forage to livestock^5^. SPS can be less detrimental to ecological health by supporting biodiversity, carbon sequestration, and water quality^6^. From a socio-economic perspective farmers also benefit from secondary forest products, such as lumber, food, medicines, and marketable fruits^7,8^. Livestock welfare benefits, in the form of limited livestock weight loss during the dry season, have also been identified which sustain milk and meat production when compared to similar systems which do not integrate silvopastoral approaches^9–12^. A number of studies have also found these benefits will lead to increased financial resilience, as costs are reduced^12–14^.

Despite these benefits, SPS have not been widely adopted in key areas of the agricultural frontier in the Amazon. Cattle ranching livestock systems still dominate the Amazonian foothills of Colombia^15,16^. Since the 2016 peace agreement a number of studies have argued that the withdrawal of the FARC (Fuerzas Armadas Revolucionarias de Colombia) has increased land and tenancy speculation, natural resource extraction, and the expansion of the agricultural frontier^17,18^. Accordingly, the conversion of native forest to cattle ranching is often facilitated by non-legal actors and land speculation^19,20^.

Context dependant factors pervade discussion of limits to adoption of SPS. The purpose of this paper is to provide a detailed examination of factors found for SPS adoption and apply these to the region of Caquetá which has one of the highest deforestation rates in the Amazon basin^21^. Echoing the conceptual framework of^22^, we categorise adoption-related factors into five distinct factors: biophysical factors, production and social factors, economic factors, farmer perceptions, and information and education related factors. Variables, nested within their respective categories, are tested against a binary adoption indicator^23^. This combined approach not only reinforces the frameworks utilised in previous studies but also introduces a novel perspective for assessing livestock-forestry adoption in the unique context of the Colombian Amazon.

**Figure 1:**
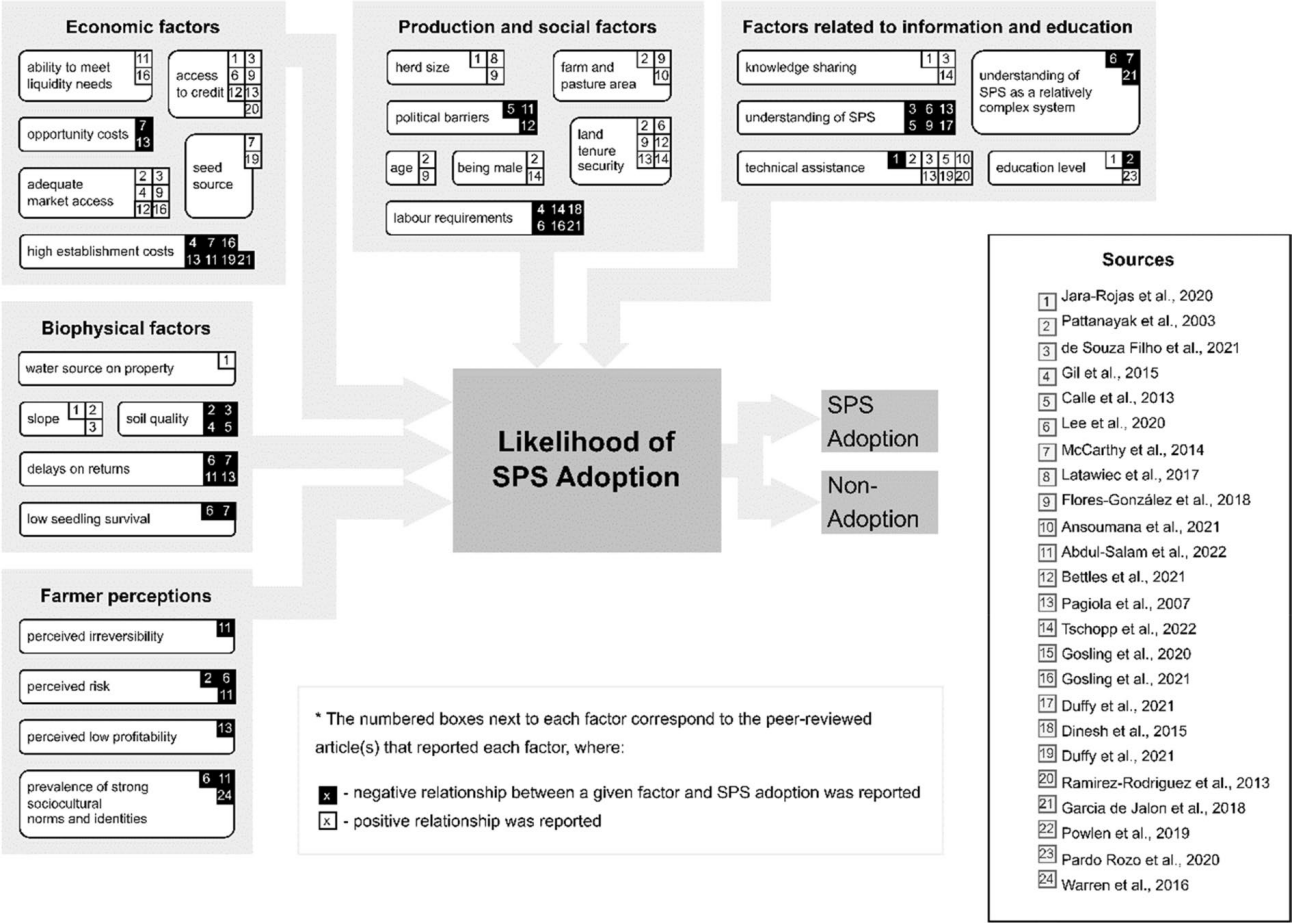
Flow chart of factors determining and inhibiting adoption of SPS We assign the general influence of these factors against the authors, where the colour-coding of the citation numbers indicate a positive or negative relationship for the adoption of SPS. Arrow thickness is arbitrary and not reflective of variable importance.

The paper is structured as follows. A conceptual background is presented which summarises the significant number of factors explored by past studies on SPS adoption. We then set multiple hypotheses based on these studies and test these through a bespoke survey of farmers within this region with the aim of comprehensively exploring each barrier. Results are presented with the aim of testing the key drivers for adoption. This is followed by a discussion of the results and implications for interventions to support transition to SPS. There is a substantial and growing literature on SPS adoption barriers in tropical agriculture. Figure 1 summarises these studies across various contexts, and they identify a range of biophysical, economic, social-cultural and perceptual factors.

Figure 1 and the accompanying literature provides the basis for a series of individual hypotheses. These are listed in Table 1 and specify separate components of the adoption problem around SPS. Steep slopes (H1) and unfavourable soils (H2) were reported to positively influence adoption^11,24^. Herd size (H3) and farm size (H4) are wealth proxies that indicate the ability of farmers to overcome establishment costs of SPS^25,26^. Capacity is also addressed through labour availability (H5; H6) and these farms are better able to overcome the high labour demands of SPS^27^. Gender has been found to be important and, due to societal gender inequalities, women may face extra barriers to adoption compared to men^22,23^. Length of farming experience tends to lead to more SPS adoption (H8;H9) and age has been found to be a significant factor on adoption (H10)^22,27^. Land tenure security is an important precursor to any type of long-term investment in the land which affects adoption decisions (H11) since there would be little incentive to invest in SPS implementation without tenure^23,28,30^. Market access is a well-documented determinant of adoption and has been proxied by several different variables, including the distance to a municipal centre (H12) or to a main road (H13). The opportunity costs of implementing SPS (H14) are higher when household members have off-farm incomes^6,22,28,29^.

**Table 1:** Description of Hypotheses.

Biophysical factors
H1. Farms in regions with a steep slope exhibit higher adoption rates than those in shallow sloping regions
H2. Farms located in veredas* with better soil quality exhibit higher adoption rates than farms in regions with less sandy soils
Production and social factors
H3. SPS are more likely to be adopted by farms with larger herds
H4. SPS are more likely to be adopted by larger farms
H5. SPS are more likely to be adopted by farms with more available household labour
H6. SPS are more likely to be adopted by farms with more available hired labour
H7. SPS are more likely to be adopted by male farmers
H8. Farmers with more years at current farm will adopt SPS
H9. Farmers with more livestock experience will adopt SPS
H10. SPS are more likely to be adopted by older farmers
Economic factors
H11. SPS are more likely to be adopted by owner-occupiers
H12. SPS are more likely to be adopted by farms with better market access, where market access is proxied by: proximity to municipal centre
H13. SPS are more likely to be adopted by farms with better market access, where market access is proxied by: proximity to a main road
H14. SPS are less likely to be adopted by farmers with off-farm jobs
Farmer perceptions
H15. SPS are more likely to be adopted by farmers who perceive the benefits to profitability
H16. SPS are more likely to be adopted by farmers who perceive the benefits to pest reduction
H17. SPS are more likely to be adopted by farmers who perceive the benefits to product quality
H18. SPS are more likely to be adopted by farmers who perceive the benefits to cost reduction
H19. SPS are more likely to be adopted by farmers who perceive the benefits to milk production
H20. SPS are more likely to be adopted by farmers who perceive the benefits to cattle reproduction
Factors related to information and education
H21. SPS are more likely to be adopted by farmers when other SPS-adopters are in their vicinity
H22. SPS are more likely to be adopted by farmers who have been trained in SPS
H23. SPS are more likely to be adopted by farmers who have completed secondary school
H24. SPS are more likely to be adopted by farmers who are members of a farmer association
H25. SPS are more likely to be adopted by farmers are confident in SPS*
H26.SPS are more likely to be adopted by farmers who have skills needed for SPS
H27.SPS are more likely to be adopted by farmers who have the ability to explain SPS

* In Colombia, “veredas” are the smallest type of subnational boundary and are spatially equivalent to a sub-municipality or neighbourhood.

Farmer perceptions are particularly crucial with respect to adoption of SPS practices. In particular, economic perceptions^6,22,30^ (H15; H18), as well as perceived wider benefits on production and welfare of cattle (H16; H17; H19; H20).

Knowledge sharing influences adoption, especially through neighbouring farmers^23^, and this effect is, presumably, amplified when the neighbours are SPS-adopters (H21). Adoption is positively influenced by training in SPS provided by organizations that promote agroforestry since they close the knowledge gaps that impede adoption (H22)^2,11,26,30^. Farmers who have completed secondary school are more likely to understand the underlying concepts of SPS and are therefore more likely to adopt (H23)^16,22^. Membership in farmer’s associations influences community knowledge sharing and has been found to have a positive influence on adoption (H24)^11,23,24^. Hypotheses H21-H24 lead to proxies for perceived understanding and confidence of SPS (H25), have the skills to implement SPS (H26) and the ability to explain SPS (H27) ^6,22,28,31^.

## Results and discussion

The results are shown in Table 2. We find no significant association between SPS and slope (H1) and soil order (H2) for these farmers in Caquetá. This is converse to previous studies^11,13,24^. Slope and soil are expected to be context dependant, and this may be the case here. Moreover, we examined soil order, rather than quality or sand proportion as used in previous studies, hence this may provide another dimension to understanding how soil influences SPS adoption.

**Table 2:** Summary of results, strength of effects and p-values for each hypothesis.

	Non-SPS		SPS		Univariate analysis		
	Mean	SD	Mean	SD	Coeff/Chi^2^	SE	*P*
H1. Slope in vereda	8.71	(4.09)	9.00	(4.32)	0.02	(0.04)	–
H2. Soil type:	3.13	(0.96)	3.48	(0.84)	7.79		–
% of and-oxisol soil	8%		5%				
% of entisol soil	15%		8%				
% of ultisol soil	33%		22%				
% of inceptisol soil	44%		65%				
H3. Herd size (no)	38.95	(45.58)	27.47	(40.35)	− 0.01	(0.00)	–
H4. Farm size (ha)	138.43	(144.92)	109.21	(119.72)	0.00	(0.00)	–
H5. Household labour (no)	2.28	(1.24)	1.82	(1.23)	− 0.30	(0.13)	*
H6. Hired labour (no)	2.28	(1.34)	2.11	(1.23)	− 0.07	(0.13)	–
H7. Male farmers (%)	87%		74%		4.54		*
H8. Livestock experience (yrs)	23.05	(9.82)	16.19	(7.31)	− 0.13	(0.03)	***
H9. Experience at current farm (yrs)	22.24	(7.99)	15.23	(6.45)	− 0.09	(0.02)	***
H10. Age (%tage over 50)	31%		24%		1.04		
H11. Ownership	83%		88%		2.39		–
H12. Proximity to municipal centre (km)	7.61	(4.64)	9.43	(5.91)	0.06	(0.03)	*
H13. Proximity to a main road (km)	5.24	(6.9)	6.84	(6.31)	0.04	(0.02)	–
H14. Off-farm job	14%		11%		0.34		–
Perceive the benefits of SPS to:							
H15. Profitability	30%		51%		9.41		**
H16. Pest reduction	19%		62%		37.92		***
H17. Product quality	44%		67%		12.06		**
H18. Cost savings	17%		72%		58.62		***
H19. Milk production	19%		72%		57.46		***
H20. Cattle reproduction	17%		71%		58.94		***
H21. More than 1 SPS in their vicinity	0%		36%		37.82		***
H22. Trained in SPS	64%		79%		4.82		*
H23. Completed secondary school	24%		13%		2.95		–
H24. Member of a farmer association	40%		29%		1.96		–
Farmers who understand SPS, indicated by:							
H25. Have confidence to implement SPS*	20%		81%		65.86		***
H26. Has skills needed for SPS^	14%		62%		55.35		***
H27. Ability to explain SPS ~	13%		60%		55.27		***

Sig. * < 0.05, ** < 0.01, *** < 0.001.

*Relates to the statement ‘I am confident that I could use different silvopastoral practices in my farm if I wanted to’.

^Relates to the statement ‘I have the skills, experience, and knowledge required to use silvopastoral practices in my farm’.

~Relates to the statement ‘I could clearly explain to other farmers the impact that the use of silvopastoral systems has on the farm’.

Despite gender being only weakly significant (H7), we find a higher proportion of female head of households will adopt SPS. Nevertheless, the majority of adopters are mostly male. Previous studies have argued that due to gender inequalities women have less access to credit, income, and equipment and this acts as a barrier to adoption to SPS^22,23,32^. The roles that women hold in Latin American cattle ranching operations are often discounted^33^ therefore, their contributions to various aspects of cattle management, such as milking, albeit significant, are often overlooked.

Some studies found length of experience to positively affect adoption of SPS, albeit with low or no statistical significance^22,27^, whereas we find the converse (H8, H9). Those variables related to experience (years on current farm and livestock experience) showed significant and negative associations with adoption, meaning that increased experience of agricultural activity decreases the likelihood of adoption. This aligns with literature on the role of farming experience which locks farmers into productivist practices compared to investment in alternative systems such as SPS^34,35^. According to systems thinking^36^, paradigms, such as embeddedness in a productivist mindset, are the intervention points in a system that are the most resistant to change but yield greater results in application. Therefore, if the negative association between experience and SPS adoption in Caquetá is a result of productivist paradigms, addressing these paradigms could generate substantial increases in adoption rates.

Access to markets has been found to be a driver for agricultural intensification^19^ but also for SPS adoption^11,37,38^ and proximity to main roads act as a proxy for this market access (H13). Here we find this is not significant and proximity to municipal centre, another indicator of market access, to be weakly associated with SPS (H12). Farmers adopting SPS are more distant from the municipal centre than non-SPS adopters. Road development is a well-known driver of deforestation both in the Amazon and internationally, therefore this approach more likely leads to adverse impacts on forest ecosystems^17,39^. Perhaps a more important limiter of market access is the lack of local markets resulting from the low population density observed in forest frontier areas within Caquetá that results from the low labour demands of extensive traditional ranching^38,40^. SPS has been found to support sustainable and profitable livelihoods in the Colombian Amazon^41^ therefore, if the other barriers to SPS are dismantled to the point where adoption becomes widespread, the concentration of people seeking SPS-based livelihoods would contribute to increasing population density and the subsequent revitalisation of local markets. Non-state actors have an advantage compared to centralized governmental programs in addressing issues at a highly local scale, for example by helping farmers to overcome regulatory market barriers^37^.

All of the six variables which reflect farmer perceptions (H15–H20) exhibited highly significant and positive associations with SPS, highlighting the importance of exploring perceptions towards the benefits of SPS. These include both perceptions of economic factors, such as yield and profits, but also pest management and cattle reproduction^37,42^. Changing perceptions would be a key route to adoption and several mechanisms, such as information exchange and education have been proposed to raise awareness of SPS in these farming populations^11,24,43^.

Another significant positive effect on adoption was farmers’ proximity to other adopters (H21). Where there are existing silvopastoral farms in veredas farmers are more likely to adopt SPS. This is a result of community knowledge sharing, a commonly reported determinant of adoption across Latin America^11,24^. A similar proximity effect was found in Argentina^23^. This suggests a spatial effect in which intra-vereda knowledge exchange occurs.

Specialised SPS training is positively associated with adoption (H22). The training of farmers in SPS is a strategy commonly suggested in the literature for raising adoption of SPS^2,24,26,31,44^. Like the perception of benefits, the understanding and confidence in SPS (H25–H27) exhibited slightly significant positive effects on adoption. Adoption was higher among farmers that either had confidence in their ability to implement SPS, had skills needed for SPS, felt that SPS were understandable, or were able to explain SPS to other farmers. The absence of knowledge gaps—in other words, the understanding of SPS—is a strong and significant determinant of adoption^27,30,31,37^. Both governmental and non-state actors, it has been argued, can contribute to closing these knowledge gaps via marketing, workshops on SPS, and specialised extension services^37^.

## Discussion

Colombia in the post-agreement landscape has experienced a range of demands on its future land use with strong climate commitments that support zero deforestation^45^. Silvopastoral systems support a transition to low carbon production but only if they intensify in sympathy with the needs of biophysical and socio-economic contexts^46,47^. Managing this transition requires locally targeted solutions and, in providing an overview of these key constraints to uptake, we find that adoption of SPS is context specific. A number of common factors associated with supporting uptake of this practice were not found to be applicable in the Caquetá region of the Colombian Amazon.

A key factor of concern is the role of increasing market access which has been found to be both a driver for deforestation but also for SPS in previous studies. In our context we find further distance to market leads to more SPS adoption and argue for establishment of local markets to support this practice. However, if the complexities between economic growth and the intensity of activity and adoption of SPS are not actively managed then this leads to a false pathway for sustainable development, or worse a potential increase in deforestation^30,47,48^.

A positive determinant for adoption is perception of the benefits and the level of understandability of farmers to the SPS system. There will be underlying causes of these perceptions which potentially lie in historic interventions and past engagement with individuals and agencies^49^. Whilst we offer a schema for understanding adoption we consider these factors in isolation to explore their association with adoption and not their causality. It is notable that studies on this topic tend to ignore the underlying causal dynamics of these factors and there are a paucity of studies examining the antecedents of these factors and their instrumentality in forming these perceptions. This is mostly a result of cross-sectional exercises in data collection and the true dynamics of these systems need to be explored further to avoid perverse outcomes from policy prescriptions.

## Methods

**Figure 2:**
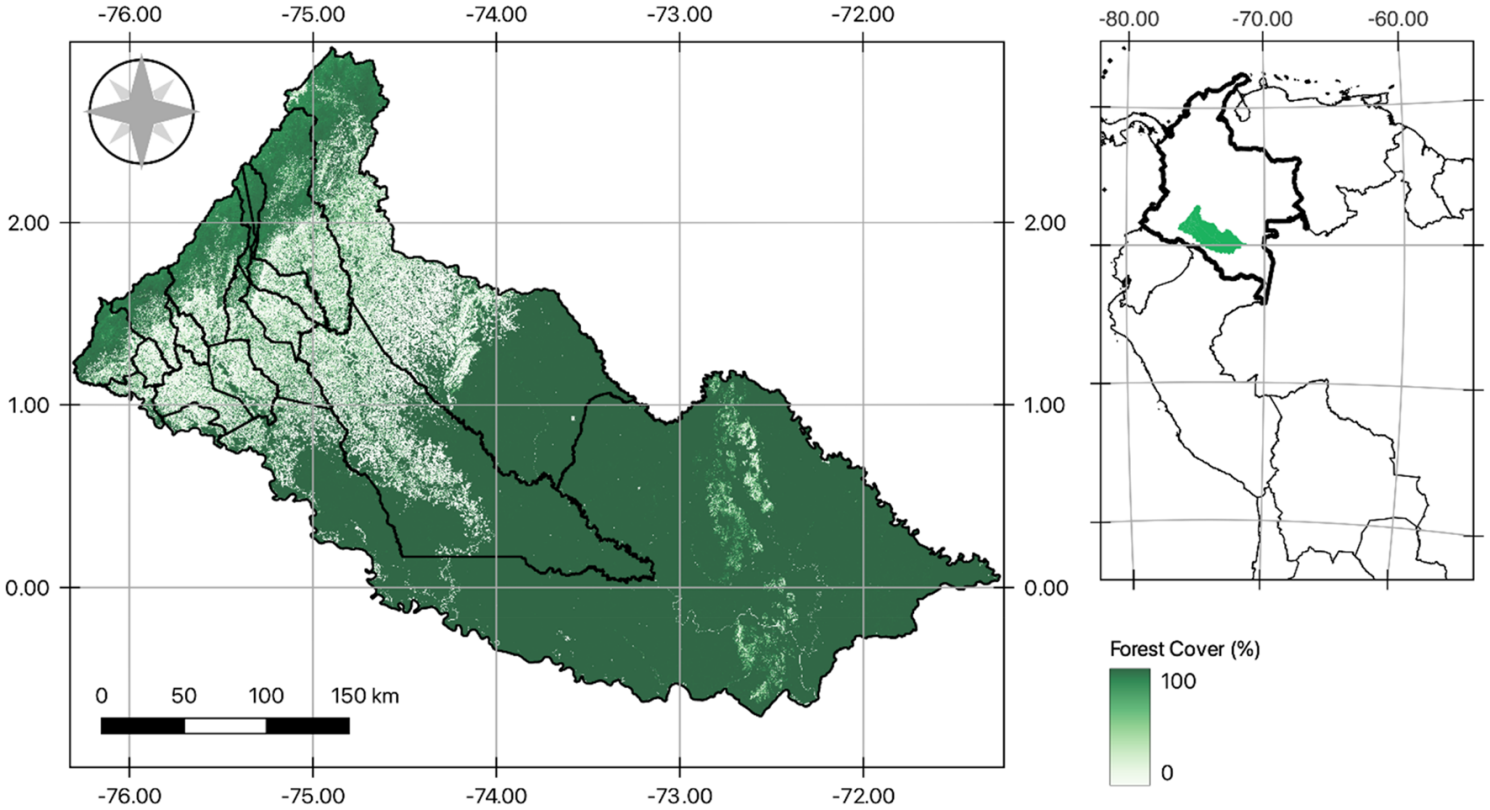
Department of Caquetá, its position within Colombia and the level of forest cover. Author’s elaboration from The Global Land Analysis and Discovery (GLAD) laboratory at the University of Maryland, in partnership with Global Forest Watch (GFW). Available at: https://storage.googleapis.com/earthenginepartners-hansen/GFC-2022-v1.10/download.html.

The Department of Caquetá covers an area of around 89 thousand km^2^ (@8% of total Colombian area) and has a variety of cropping and livestock activities. It is the third largest department in Colombia but with low levels of population density. As it sits within the Amazon basin Caquetá has highly important and rich ecological diversity and has a high density of forest cover (Fig. 2). Given it remoteness and position it was heavily affected by the armed conflict and has been the focus for investment and infrastructure support in the post-agreement landscape^50^. Critical land use pressures occur from the illegal cultivation of coca in the region but also mineral and fossil-fuel extraction. Moreover, nearly 60% of rural land in Caquetá is legally informal or imperfect which creates limits on accessing institutional support regimes^51^.

Caquetá is Colombia’s fifth largest milk producer which is characterised by smallholder extensive cattle farming. Due to its medium altitude farmers tend to adopt mixed systems of dairying and beef farming with tropically adapted breeds crossed with dairy breeds and yields are low relative to more intensive regions^52^. Agriculture provides the main source of income for local livelihoods and mostly a source for exports for the Colombian economy^53^.

The sampling universe was compiled by working with the local department of Agriculture in Caquetá, with local producers’ associations and companies that purchase milk from these producers. This led to an overall sample of 1100 registered farmers in the region, with 112 who were previously identified to have imposed silvopastoral systems on farm. Detailed information was received from companies such as Nestlé, Alimentos GAMAR, the Ministry of Agriculture's Milk Price Monitoring Unit and departmental agricultural leaders. The farm database was created to mobilise a telephone-based survey. This was favoured due to issues around remoteness and accessibility and collating a large enough sample to conduct robust statistical tests. Whilst this imposes some bias, e.g., to larger operations, mobile phone usage is fairly common in the Caquetá region, with farmers using mobile phones as part of their business operations^54^. Farmers were told their participation was voluntary and information that may identify them would only be held on a secure server and not shared with third parties. Structured phone interviews were conducted with farmers across the study area with the aim of collecting an equal sample between adopters and non-adopters across the region. As a result, 172 farms were selected such that 86 (50%) had adopted silvopastoral systems on at least one hectare of land, and the other half had not.

Once completed these data were matched through GPS co-ordinates, located at the centre point of each corresponding vereda, to geospatial variables that had been aggregated to the vereda level using the mean. The spatial variables, which were derived from online sources, were soil type and slope. Soil data was obtained from the website of the Instituto Geografico Agustin Codazzi (IGAC)^55^. Slope data was derived from a global digital elevation model^56^.

### Statistical analysis

We employ a univariate approach to test our hypotheses and identify their association with SPS adoption^11^. We applied Pearson’s chi-square test of independence in categorical variables, and logit regression where the explanatory variable is continuous. These were assessed against the binary adoption variable of adoption or non-adoption of SPS^22,23^. The categorical variables fulfilled the requirements of the Pearson’s chi-square test, including independence, mutual exclusivity, and expected values of five or more in at least 80% of the contingency table cells^57^. Logistic regression results are presented as log-odds as a change in the independent variable for predicting the dependant variable.

## Data Availability

Anonymised data and codes are available on reasonable request from Andrew.Barnes@sruc.ac.uk.
